# Improved Algorithm for Insulator and Its Defect Detection Based on YOLOX

**DOI:** 10.3390/s22166186

**Published:** 2022-08-18

**Authors:** Gujing Han, Tao Li, Qiang Li, Feng Zhao, Min Zhang, Ruijie Wang, Qiwei Yuan, Kaipei Liu, Liang Qin

**Affiliations:** 1 Department of Electronic and Electrical Engineering, Wuhan Textile University, Wuhan 430200, China; 2 State Grid Information & Telecommunication Group Co., Ltd., Beijing 102211, China; 3 School of Electrical and Automation, Wuhan University, Wuhan 430072, China

**Keywords:** aerial insulator images, object detection, YOLOX, small target, SIoU

## Abstract

Aerial insulator defect images have some features. For instance, the complex background and small target of defects would make it difficult to detect insulator defects quickly and accurately. To solve the problem of low accuracy of insulator defect detection, this paper concerns the shortcomings of IoU and the sensitivity of small targets to the model regression accuracy. An improved SIoU loss function was proposed based on the regular influence of regression direction on the accuracy. This loss function can accelerate the convergence of the model and make it achieve better results in regressions. For complex backgrounds, ECA (Efficient Channel Attention Module) is embedded between the backbone and the feature fusion layer of the model to reduce the influence of redundant features on the detection accuracy and make progress in the aspect. As a result, these experiments show that the improved model achieved 97.18% mAP which is 2.74% higher than before, and the detection speed could reach 71 fps. To some extent, it can detect insulator and its defects accurately and in real-time.

## 1. Introduction

Insulators are key components that provide electrical insulation and mechanical support for current-carrying conductors on high-voltage transmission lines. Defects are likely to occur due to various factors such as transient loads, mechanical stress, atmospheric conditions, etc. Furthermore, they might then threaten the stable operation of transmission lines which highly impacts the security of the power system. A UAV (Unmanned Aerial Vehicle) is more efficient and convenient as it offers visual assessments of structures. Therefore, it has gradually replaced the traditional manual inspection method. The detection of insulator defects based on aerial images consequently has become popular. However, insulator defects in aerial images often exhibit small targets and complex backgrounds in the dataset. Therefore, it is difficult to detect insulator defects quickly and accurately.

Traditional methods of insulator defect detection focus on color, texture, edge, and other features [1,2,3,4]. This kind of method relies on high-quality images and appropriate shooting angles. It might suffer from weak robustness.

Object detection algorithms based on deep learning have been widely used in power systems due to the good performance of the generalization capability and the ability to extract features from complex backgrounds [5,6]. They are generally divided into two categories: one-stage algorithm and two-stage algorithm. These two-stage object detection algorithms mostly use RPN (Region Proposal Network) to reduce the interference of complex background on insulator and its defect detection. Although the precision of insulator detection could be improved, the low efficiency and slow speed cannot be ignored [7,8,9,10,11,12,13,14,15].

The YOLO series is a typical one-stage object detection algorithm, which eliminates the RPN and generates the position coordinates and category probability of the object through a single detection, which can quickly and accurately complete the detection task [16]. Since its inception, the YOLO series of the algorithm is gradually developed in terms of accuracy and speed. Moreover, it has evolved a variety of more advantageous algorithm models such as YOLOv3, YOLOv4, YOLOv5, and YOLOX. In terms of the application in defect detection, Wang et al. [17] used Gaussian parameters to model the coordinates of the predicted box, which improved the accuracy of the YOLOv3 algorithm to detect defects to some extent. Zhang et al. [18] used the YOLOv3 algorithm with a dense FPN structure to improve the utilization of deep semantic information and shallow localization information, reduce the number of model parameters, and improve the detection accuracy of insulator defects. Shen et al. [19] and Duan et al. [20] suggested that the defect detection accuracy of the YOLOv3 algorithm could be improved by optimizing the regression loss. Tang et al. [21] divided the task of defect detection into two parts and improved the accuracy by using YOLOv4 to detect defects in insulators segmented by U-net. Lv et al. [22] investigated the effect of clustering algorithms on the detection results of the model, studied the effect of regression loss on targets at different scales, and proposed an intelligent identification method for electrical devices based on the YOLOv4 algorithm. Qiu et al. [23] used depthwise separable convolution to reduce the number of parameters of the YOLOv4 algorithm, improved the detection speed of the model, and used the Laplace sharpening method to preprocess the insulator image. It actually alleviated the problem of reduced detection accuracy caused by model lightweighting. Moreover, these studies are mainly based on YOLOv4 or earlier versions, and there are few studies about the regression perspective of regression loss on model accuracy.

Compared with the above algorithms, YOLOX has a faster detection speed and higher accuracy on the COCO dataset [24]. The detection speed of its lightweight model YOLOX-S achieved 75 fps which could make progress in the speed of defect detection. However, this model still suffers from the problem of low accuracy when it detects defects. At the same time, its detection results are vulnerable to the influence of complex backgrounds.

To improve the efficiency and accuracy of detection of insulator defects in high-voltage transmission lines, this paper discusses the problem that defects are difficult to detect and optimizes the YOLOX algorithm by further researching the regression loss. As for complex backgrounds, the influence of the attention mechanism on the model accuracy is also considered.

This paper proceeds as follows: It researches and analyses the defects of the regression loss of the model, considering the shortcomings of this regression loss function and further studies the law of the influence of regression angle on the accuracy of the model. The effects of different attention mechanisms on the detection effect of the model are analyzed. An improved YOLOX-S-based insulator defect detection method is proposed to achieve better results without almost changing detection speed.

## 2. Structure and Characteristics of the YOLOX-S Model

**Figure 1:**
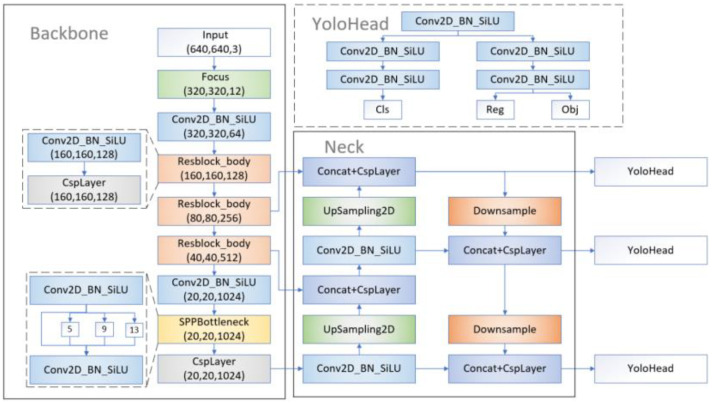
Model structure of YOLOX.

The structure of the YOLOX-S model is shown in Figure 1, which can be divided into three parts: Backbone (feature extraction network), Neck (feature fusion network), and Head (prediction network).

The Backbone including CSPDarknet performs convolution calculation on the input image, extracts sample features, and generates five feature layers containing different levels of semantic information. Finally, it selects the last three feature layers as the input information of the Neck.

The Neck part uses PANet (Path Aggregation Network) to fuse the feature information extracted by the Backbone part. Therefore, it not only contains the information of position, texture, edge, and others in low layers but also the strong semantic information in high layers.

Different from the Head of the previous models of the YOLO series, the YOLOX model decoupled the classification and the localization task. it solved the conflicts caused by the coupled two tasks and improved the convergence speed.

Since YOLOX adopts the idea of Anchor-free, it is unnecessary to preset anchor box which greatly reduces the computation of anchor-box clustering and improves the detection speed of the model. However, the model cannot effectively mitigate the influence of redundant information brought by complex backgrounds. In some special cases, the regression loss adopted by the model cannot effectively guide the regression of the model, so the detection accuracy of the model is low.

## 3. Improvements to the YOLOX Model

**Figure 2:**
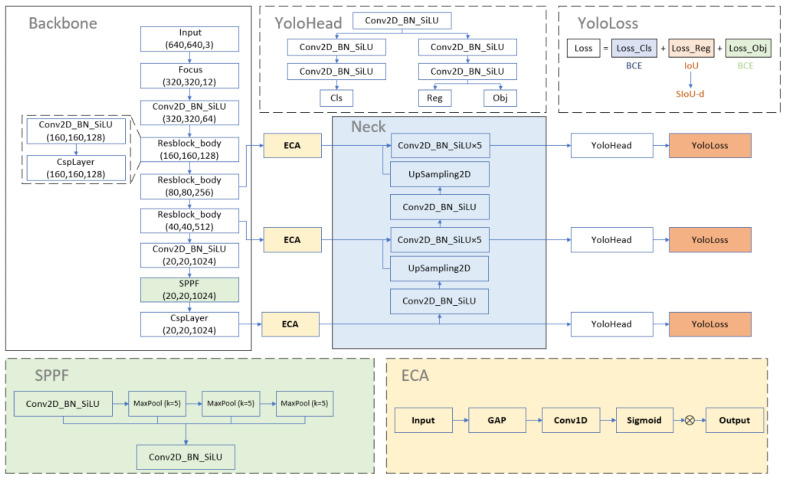
The structure of the improved model.

This section analyses the shortcomings of the regression loss used in the original model and proposes a new regression loss function, SIoU-d. Meanwhile, the structure of the model is modified by replacing SPP with SPPF which slightly reduces the computational effort. Furthermore, PAN (path aggregation network) is replaced by FPN (feature pyramid) and ECA is embedded between the backbone and feature fusion layers to lighten the effect of the complex background. Figure 2 shows the structure of the improved model.

### 3.1. IOU Loss Analysis and Improvement

Insulator defect targets are small targets with low proportion and small scale in the input image. In the actual detection process, a small amount of offset and scaling of the predicted box can make significant impact on the detection accuracy of the model for small targets. Therefore, a suitable regression loss function would be important because it could effectively optimize the regression performance of the model and improve the detection accuracy of the model for defective small targets. At present, IoU (Intersection over Union) is often used as the evaluation index of the model edge regression effect. According to it, Yu et al. [25] proposed IoU loss. Moreover, regression losses such as GIoU [26], DIoU [27], CIoU [27], and EIoU [28] were proposed in the subsequent development.

**Figure 3:**
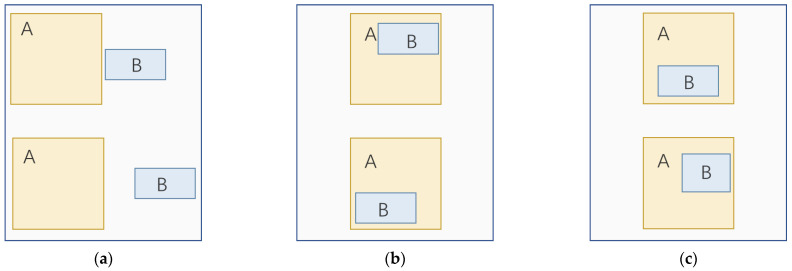
The shortcoming of IoU, A refers to the ground-truth box, and B refers to the predicted box: (**a**) the predicted box does not intersect with the ground-truth box; (**b**) the same predicted box is in different positions within the ground-truth box; (**c**) different predicted boxes with the same area in the ground-truth box.

The YOLOX-S model adopts IoU Loss as the regression loss of the model, and this loss is consistent with the evaluation index of the border regression which can guide the direction of model optimization to some extent. However, there are some problems. First of all, as is shown in Figure 3a, the two boxes have no intersection, and the value of IoU Loss is always 1, which means that it cannot effectively guide the optimization direction of the model. Secondly, when the same predicted box is in different positions within the ground-truth box, the value of IoU Loss remains the same, which cannot play a positive role in the optimization of the model, as in Figure 3b. In addition, predicted boxes of different shapes may have the same loss value within the same ground-truth box, as in Figure 3c. Finally, IoU does not specify the regression angle of the model, and the high degree of freedom in regression hinders the fast and accurate convergence of the model.

As mentioned above, Gevorgyan [29] proposed the SIoU loss function. The new penalty terms are introduced based on IoU. Firstly, the *x*-axis component and *y*-axis component of the centroid distance are compared with the width and height of the smallest external rectangle. Then, the scale-insensitive information of the centroid distance on the *x*-axis and *y*-axis would be obtained which speeds up the regression of the model and improves the regression accuracy of the model. Secondly, the angles formed by the centroids of the two boxes and the x and y axes are calculated, and the angle loss is used to guide the centroids of the predicted boxes to regress along the x and y axes of the ground-truth box centroids. It reduces the freedom of the regression and further accelerates the convergence of the network. Finally, the width and height of the two boxes were compared separately. The scale-insensitive information of width and height was obtained, and it could further improve the regression accuracy of the model.

The SIoU performs in the optimization process of the predicted box with the center point of the predicted box converging to the *x*-axis. To be specific, it adjusts the center point of the predicted box approximately to the *x*-axis with the center point of the ground-truth box and further reduces the distance components of the two center points in the *x*-axis direction when the shapes of the two boxes are similar. In this process, the width and height of the predicted box and the distance components of the two center points in the *y*-axis direction are continuously adjusted to make the predicted box coincide with the actual box as much as possible.

**Figure 4:**
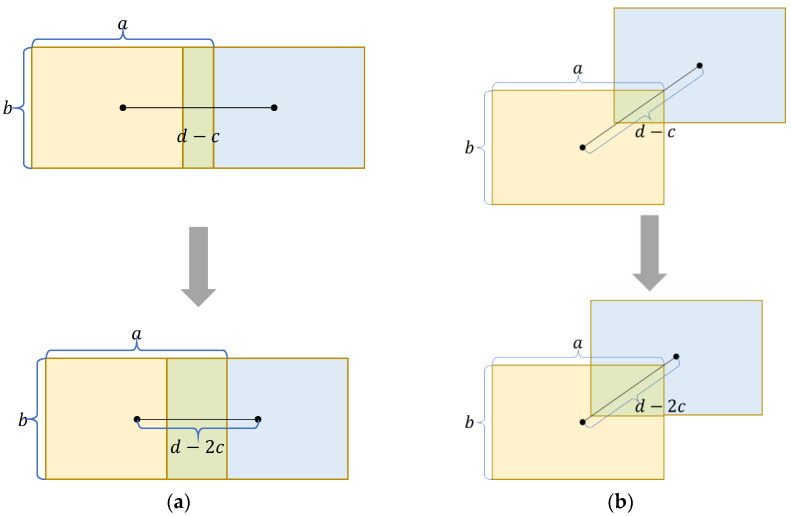
The yellow box refers to the ground-truth box, the blue box refers to the predicted box, the green box refers to the area where two boxes coincide. (**a**) The predicted box converges along the *x*-axis direction to the ground-truth box; (**b**) the predicted box converges along the diagonal direction of the ground-truth box to the ground-truth box.

Figure 4 shows the different convergence of the predicted box to the ground-truth box along the *x*-axis direction and the diagonal direction of the ground-truth box, respectively. In both figures, the width of the predicted box and the ground-truth box are *a*, the height is *b*, the original distance between the center points of both boxes is *d,* and the convergence rate is *c*. Figure 4a shows the convergence of the predicted box to the ground-truth box along the *x*-axis direction. The predicted box converges to the ground-truth box in two consecutive times, and the increment of the degree of overlap between the two boxes is *b × c*. Figure 4b shows the convergence of the predicted box to the ground-truth box along the diagonal direction of the ground-truth box, and the increment of the degree of overlap of the two boxes is growing in the course of the two successive converged actual boxes. The increment of the degree of overlap is $\frac{abc^{2}}{a^{2}+b^{2}}$ in the first convergence process and $\frac{\left(3abc^{2}\right)}{a^{2}+b^{2}}$ in the second convergence process. Therefore, forcing the predicted box to converge along the diagonal direction of the ground-truth box can effectively increase the regression efficiency of the model and enable the loss function to converge quickly in the later stages of model optimization. Meanwhile, the convergence along the diagonal direction of the ground-truth box can make the center point of the predicted box on the x, y axes fall simultaneously in a certain proportion, which is more conducive to the optimization of the model.

In this paper, the angle loss of SIoU is improved, and SIoU-d is defined as follows:

(1) $$L_{SIoU-d}=1-IoU+\frac{\Delta +\Omega }{2}$$

(2) $$\Delta =2-e^{(\Lambda -2)\times {\left(\frac{c_{w}}{C_{w}}\right)}^{2}}-e^{(\Lambda -2)\times {\left(\frac{c_{h}}{C_{h}}\right)}^{2}}$$

(3) $$\Omega ={\left(1-e^{-\frac{\left|w-w^{gt}\right|}{\max(w,w^{gt})}}\right)}^{\theta }+{\left(1-e^{-\frac{\left|h-h^{gt}\right|}{\max(h,h^{gt})}}\right)}^{\theta }$$

**Figure 5:**
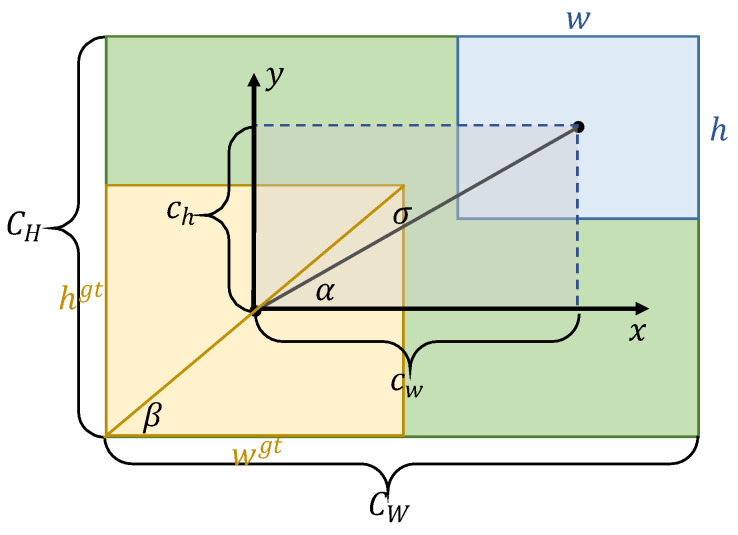
Schematic diagram of SIoU. The yellow box refers to the ground-truth box, the blue box refers to the predicted box, and the green area refers to the minimum external rectangle of the two boxes.

(4) Λ=cos(β−α)

where Δ and Ω are the distance loss and shape loss. As is shown in Figure 5, the yellow box is the ground-truth box, and the blue box is the predicted box. *c_w_* and *c_h_* are the width and height of the rectangle constructed at the center of the two boxes, *C_w_* and *C_h_* are the width and height of the minimum external rectangle, *w* and *h* are the width and height of the predicted box, and *w_gt_* and *h_gt_* are the width and height of the ground-truth box. *α* refers to the angle between the center point of the predicted box and the center point of the ground-truth box, and *β* refers to the diagonal angle of the ground-truth box.

### 3.2. Analysis of Feature Fusion and Improvement of Embedded Attention Mechanism

The backbone of the model could extract a large amount of feature information. As the depth of the model increases, the model could extract not only the shallow location features but also the deep semantic features. In the meantime, it is necessary to combine the shallow location information with the deep semantic information to enhance the detection of the model at different scales.

The YOLOX model adopts PANet as the feature fusion layer. Firstly, the semantic features of the deep layer are passed to the shallow layer by up-sampling. Then the fused feature information is passed to the deep layer by down-sampling which increases both the semantic expression capability of the shallow layer and the localization capability of the deep layer. However, this feature fusion approach might be complicated for the detection of insulator defects, so this paper uses FPN as the feature fusion layer of the model and outputs the feature information from the deep layer directly to the prediction network of the model.

However, the information extracted by the backbone contains both valid feature information and invalid redundant features. The process of feature fusion cannot effectively reduce the impact of redundant information on the detection capability of the model, while the attention mechanism can assign greater weight to important features, reduce the weight of redundant information, and lighten the influence of redundant features [30]. Therefore, it is necessary to use the attention mechanism to process the extracted feature information and assign different weights to the feature information before the model undergoes feature fusion.

The attention mechanism originates from the study of human vision, which can selectively focus on important information. Channel attentions such as ECA (Efficient Channel Attention Module) and SE (Squeeze and Excitation) could assign weights to the features of each channel, it can improve the classification ability of the model to a certain extent. Spatial attention such as Non-Local Block could assign weights to the region where the target is located which can reduce the influence of the background and enhance the regression ability of the model. Moreover, there is a mixture of the above two types of attention, CBAM (Convolutional Block Attention Module). To reduce the impact of redundant information and keep the high detection speed of the model, ECA was finally selected to enhance the weights of important features before feature fusion.

**Figure 6:**
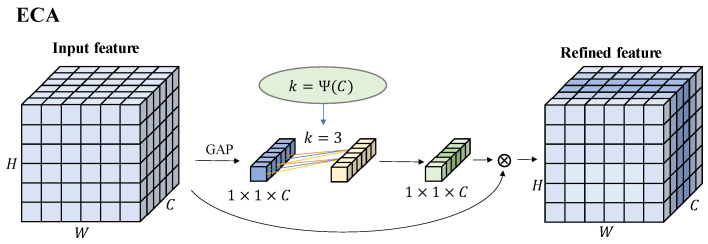
The module structure of ECA.

The structure of the ECA module is shown in Figure 6, and its generated channel weights can correspond to the channels of the input feature information which can effectively improve the learning efficiency of the model. The value of k in the figure is adaptively related to the number of channels and is defined as follows:

(5) $$k=\Psi (C)={\left|\frac{\log_{2}(C)+b}{\gamma }\right|}_{odd}$$

where *C* represents the number of channels of the input feature information, and *k* is the nearest odd number of *C* which has been processed, *b* = 1, *γ* = 2.

## 4. The Experiment and Evaluation Indexes

### 4.1. Experimental Conditions

Experiments were performed on a local Ubuntu 20.04.2 computer with 96 GB memory, CPU (Intel Xeon Platinum 8171M@ 2.60 GHz), 2 GPUs (NVIDIA GeForce RTX3090, 24 GB), and environment version is Pytorch 1.6.0.

This experiment used 1588 aerial images of insulators, including 647 images with defects. There were 2908 insulators in total and 715 insulators with defects. In the experiment, the images of the entire data set were scrambled, and the data set was divided into the training set, validation set, and test set according to the ratio of 8:1:1. Finally, 1286 images of the training set, 143 images of the validation set, and 159 images of the test set were obtained. The number of insulators and defects in each set is shown in Table 1.

**Figure 7:**
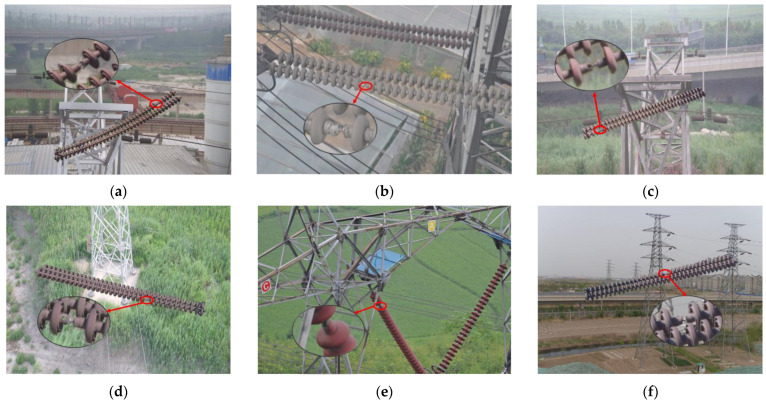
Images (**a**–**f**) show the defect of insulators.

The aerial images which have the defect of insulators are shown in Figure 7.

### 4.2. Evaluation Indexes

In this experiment, three indicators of *mAP* (mean Average Precision), FPS (Frames Per Second) are used to evaluate the model. *AP* refers to the area of the curve enclosed by the prediction accuracy and recall of the model for a certain type of target. the definitions of *AP* and *mAP* are

(6) $$AP=\int_{0}^{1}P(R)dR$$

(7) $$mAP=\frac{\sum_{i=1}^{k}AP_{i}}{k}$$

*P*(R) refers to the Precision–Recall Curve, *k* represents the number of classes. Equations (8) and (9) show the calculation of precision and recall.

(8) $$Precision=\frac{TP}{TP+FP}$$

(9) $$Recal=\frac{TP}{TP+FN}$$

*TP* (True Positive) is the example that represents the positive sample that is correctly classified. *FP* (False Positive) means the negative sample that was misclassified. *FN* (False Negative) is the example that represents a misclassified positive sample.

### 4.3. Experimental Process

The experiments adopt the idea of transfer learning to train the model with pre-trained weights for 300 epochs. The first 50 epochs freeze the backbone of the model with the initial learning rate set to 1 × 10^−3^ and the batch size set as 16. After 50 epochs, the whole model is trained with the initial learning rate reduced to 1 × 10^−4^ and the batch size set as 8. To facilitate the comparison of the effects of different improvements on the experimental results, only one variable was changed for each training. The variables of the study included the regression angle of regression loss and the attention mechanism (SE, ECA, CBAM, and Non-Local).

**Figure 8:**
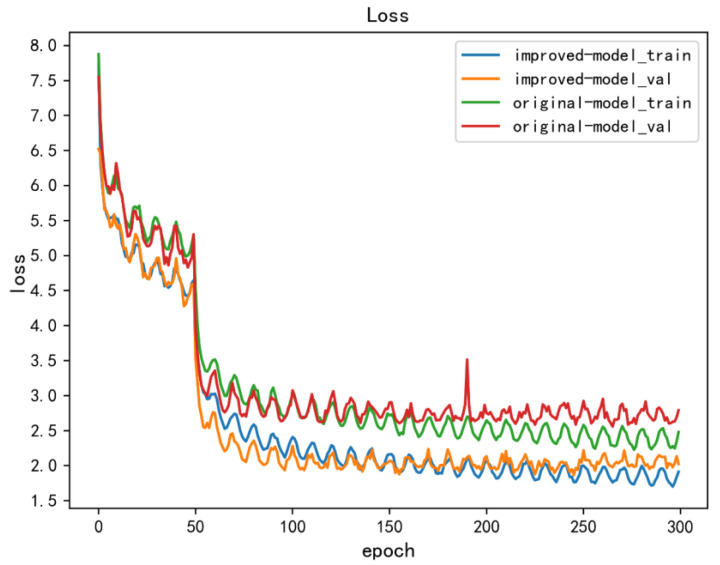
Loss curves of the original model and the improved method.

Figure 8 shows the loss curves of the original YOLOX-S model and the improved model in this paper. In the figure, the validation loss of the original model converged to 2.6, and the validation loss of the improved model converged to 1.9. The trends gradually became stable in the subsequent training processes without overfitting.

## 5. Research on Model Optimization Methods

### 5.1. The Effect of the Angle of Regression Loss

**Figure 9:**
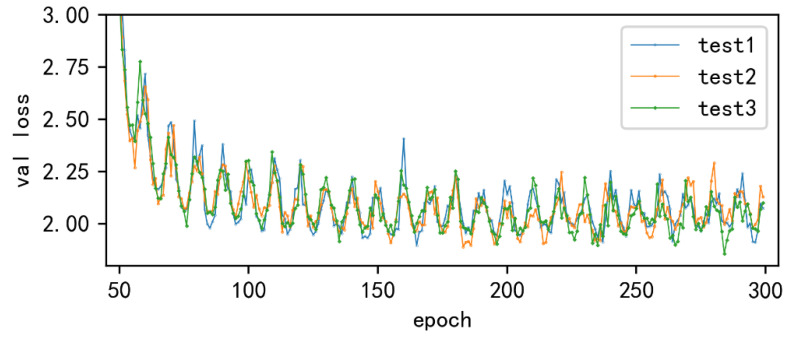
Validation loss of training with different regression angles.

The regression angle is defined as the angle between the center point of the predicted box and the center point of the ground-truth box. Figure 9 illustrates the validation loss of the model with different regression angles. Test1 defines the regression angle as the diagonal angle of the ground-truth box, so that the center point of the predicted box regresses along the diagonal direction of the ground-truth box. Test2 defines the regression angle as ^π^/_4_, so that the center point of the predicted box regresses to the center point of the ground-truth box along the line where ^π^/_4_ is located. Test3 defines the regression angle as 0, so that the predicted box is regressed along the *x*-axis and *y*-axis direction where the center point is located.

During the training of test1, test2, and test3, the number of training epochs, in which the validation loss of training is less than 2, for the first time is 85, 114, and 116, respectively. It is concluded that the test1 has the fastest loss decrease and better training effect during the training.

Table 2 shows the detection accuracy of the models trained with these three regression angles with AP50. Test3 has the lowest detection AP for both targets. Test2 has the highest detection AP for defects, but it reduces the AP of the model to detect insulators. Test1 has a slightly lower detection AP than test2 for defects while it has the largest mAP, 94.51%. Overall, test1 performs the best with improved detection AP for both targets. Compared with the original model, the detection AP for defects is increased by 2.74%, and the detection AP for insulators is increased by 0.18%.

The experimental results show that the closer the selected regression angle is to the angle of the diagonal of the ground-truth box, the faster the convergence of the model, the higher the detection accuracy. Therefore, the angle of the diagonal of the ground-truth box is selected as the angle of the model regression loss in this paper.

### 5.2. The Impacts of Attention Mechanism

To investigate the influence of attention mechanism on model detection accuracy, this paper embedded SE, ECA, CBAM, and Non-Local between the backbone and feature fusion layers of the model and trained the model separately.

Table 3 shows the detection AP of the models with these four attention mechanisms at AP50. The model detection mAP with embedded SE, ECA, CBAM, and Non-Local is 94.74%, 95.17%, 95.10%, and 94.40% where ECA and CBAM performed similarly. CBAM is slightly better than ECA in defect detection. It is mainly due to the reason that CBAM has both channel and spatial attention. Spatial attention has an enhanced effect on the regression effect of the predicted box, but this attention mechanism has a more complex computational process compared to ECA and has a greater impact on the detection speed of the model. Considering these factors, ECA has a better performance with the AP increased to 0.59% for insulators and 0.86% for defect AP. It can effectively assign weights to the feature information and improve the detection effect of the model.

**Figure 10:**
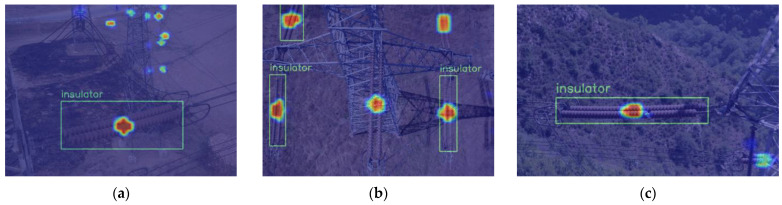
Heat map of the detection of the original YOLOX-S model: (**a**) only one insulator has been detected; (**b**) three insulators have been detected; (**c**) only one insulator has been detected, and the defect has not been detected.

**Figure 11:**
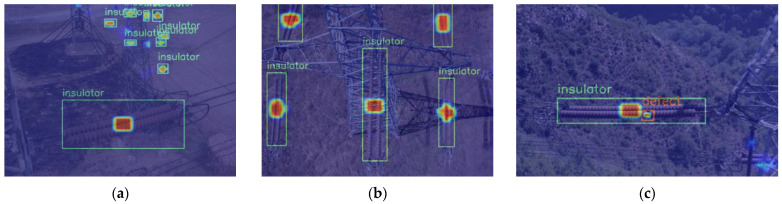
Heat map of the detection with the ECA embedded: (**a**) nine insulators have been detected; (**b**) five insulators have been detected; (**c**) one insulator and one defect have been detected.

Figure 10 shows the heat map of the original model, and Figure 11 shows the heat map of the model with ECA embedded. Among them, the center of the target which is focused by the model would be highlighted. The higher the brightness, the more attention it receives. The green box represents the insulator detected, and the orange box represents the insulator defect detected. Only some of the highlight insulators in Figure 10a,b are detected. In addition, the insulator defects in Figure 10c receive almost no attention from the model. Figure 11a,b can detect the low confidence insulators in Figure 10a,b which received attention from the model but were not detected. As for the insulator defects that existed in Figure 10c, the model pays high attention and detected them successfully. The results indicate that the embedding of the attention mechanism can effectively mitigate the influence of redundant features, improve the sensitivity of the model to important features of the target, and improve performance.

### 5.3. The Comparison of Predicted Results

As is shown in Table 4, ablation experiments are used to verify the effectiveness of the improved algorithm in this paper. The position of “√” in the table indicates that the algorithm adopts this improved strategy. Algorithm 3 introduces CBAM on the basis of Algorithm 2. This attention mechanism has little effect on the improvement of model detection AP. The detection AP of insulators is increased by 0.1%, and the defect detection AP is increased by 0.51%.

In comparison, ECA is introduced in Algorithm 4 on the basis of Algorithm 2. The introduction of this attention mechanism greatly improves the detection AP of the model for small targets with defects. The insulator detection AP is increased by 0.29%, and the defect detection AP is increased by 1.51%. This result shows that ECA has a better performance in improving the detection effect of the improved model in this paper.

Based on the above studies, this paper proposed an improved YOLOX-S model by using SIoU-d as the regression loss of the model to enhance the regression performance of the model and ECA to lighten the influences of redundant features on the detection accuracy of the model.

**Figure 12:**
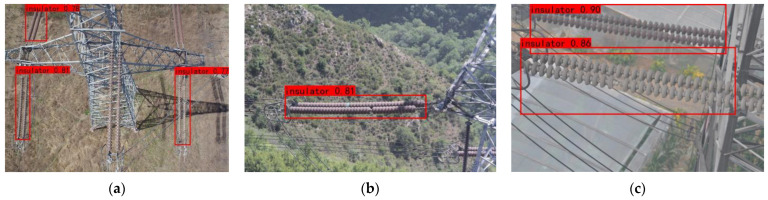
Detection result of the original model: (**a**) three insulators have been detected; (**b**) only one insulator has been detected, and the defect has not been detected; (**c**) two insulators have been detected, and the defect has not been detected.

**Figure 13:**
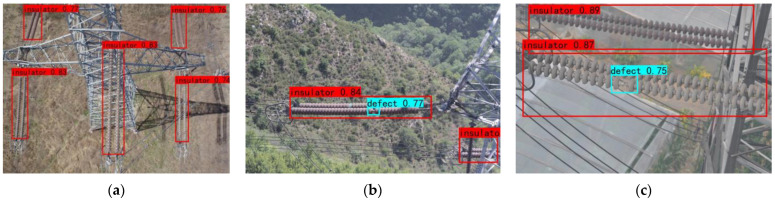
The detection result of the improved model: (**a**) five insulators have been detected; (**b**) two insulators and one defect have been detected; (**c**) two insulators and one defect have been detected.

Figure 12 shows the predicted results of the original model, and Figure 13 shows the predicted results of the improved model, where the red boxes indicate the detected insulator targets, and the blue boxes indicate the detected defect targets. Only three insulators were detected in Figure 12a. It is clear that insulators with complex backgrounds and insulators with similar colors to the background cannot be detected well. One insulator was detected in Figure 12b, and the defects present on it and the blocked insulator were not detected. Two insulators were detected in Figure 12c, but the insulator which is located below it was not completely boxed out. Figure 13a detects the two insulators that could not be detected inFigure 12a.; Figure 13b detects the defect present on the insulator and the insulator that is blocked in the lower right corner. Figure 13c detects the defect located on the insulator below while completely boxing out the insulator below.

The images adopted in the prediction of experiments are the actual detection images which were not used in the training. The results show that the improved model could effectively lighten the influence of the complex background on the detection of insulators and its defects. The model could accurately detect insulator defects and have the ability of generalization.

Table 5 shows the comparison of the improved model with other models at AP50. The Algorithm 4 achieves the best results in the detection of insulators and defective targets, with 96.57% AP for insulator detection which increased by 0.45% and 97.79% AP for defective targets which increased by 5.03%. In addition, there is no obvious decrease in its detection speed.

## 6. Conclusions

To achieve intelligent inspection of transmission lines, lighten the influence of complex background on model detection, and improve the detection effect of the model on defects, this paper explores the effects of regression angle and attention mechanism on model accuracy based on YOLOX-S. The experimental results show that the regression of the predicted box along the diagonal direction of the ground-truth box can effectively enhance the regression effect of the model and improve the detection accuracy of the model for small targets. The embedded of the channel attention mechanism between the backbone and feature fusion layers can effectively lighten the influence of the complex background on the detection accuracy of the model. The improved model in this paper has a detection AP of 97.79% for insulator defects, achieving a rise of 5.03%. In addition, detection AP reached 96.57% for insulators, a rise of 0.45%. The detection speed rose to 71 fps which can satisfy the purpose of fast and accurate detection of defective small targets.

## Figures and Tables

**Table 1:** Data set division.

Dataset	Image	Insulator	Defect
Train	1286	2346	578
Val	143	257	65
Test	159	305	72
Total	1588	2908	715

**Table 2:** Detection AP of the models trained with different regression angles at AP50.

Method	Defect AP/%	Insulator AP/%	mAP/%
Base ^1^	92.76	96.12	94.44
Test1	95.39	96.30	95.84
Test2	95.44	96.04	95.74
Test3	95.33	95.96	95.64

^1^ The base model is YOLOX-S.

**Table 3:** Detection AP of the models with different attention mechanisms at AP50.

Method	Defect AP/%	Insulator AP/%	mAP/%	Fps
SE	93.52	95.92	94.74	71
ECA	93.62	96.71	95.17	71
CBAM	93.89	96.31	95.10	66
Non-Local	93.34	95.46	94.40	33

**Table 4:** The ablation experiments of different improvement strategy.

Model	FPN	SIoU-d	ECA	CBAM	Insulator AP/%	Defect AP/%	mAP/%	Fps
YOLOX-S					96.12	92.76	94.44	74
Algorithm 1		√			96.30	95.39	95.84	72
Algorithm 2	√	√			96.28	96.28	96.28	71
Algorithm 3	√	√		√	96.38	96.79	96.58	62
Algorithm 4	√	√	√		96.57	97.79	97.18	71

**Table 5:** Detection accuracy of different models at AP50.

Model	Insulator AP/%	Defect AP/%	mAP/%	Fps
Faster-RCNN	93.24	65.05	79.15	8
SSD	86.74	62.04	74.39	65
YOLOv3	93.62	89.68	91.65	39
YOLOv4	91.86	90.43	91.15	32
YOLOv5-S	92.54	93.03	92.78	71
YOLOX-S	96.18	92.93	94.55	74
Algorithm 4	96.57	97.79	97.18	71

## Data Availability

Dataset link: https://github.com/InsulatorData/InsulatorDataSet (accessed on 18 May 2022).
